# A Large-Scale Anatomically and Electrophysiologically Constrained Model of Hippocampus at Behavioral Time Scales

**DOI:** 10.21203/rs.3.rs-11447128/v1

**Published:** 2026-10-22

**Authors:** Dong Song, Gene Yu, Chenlin Xu, Lars Niedermeier, Xiang Zhang, Hongwei Dong

## Abstract

Large-scale mechanistic brain models remain challenging to construct from heterogeneous anatomical and physiological data, simulate over behavioral timescales, and optimize against network-level activity. Here, we introduce a GPU-accelerated computational framework that transforms population-level anatomical measurements into explicit, single-neuron-resolution connectivity and integrates cell-type-specific neuronal and synaptic dynamics. A two-stage optimization strategy combines calibration against unitary synaptic responses with physiologically informed network-level optimization, using online parameter updates during continuous simulation. We demonstrate the framework in a hippocampal network comprising approximately 250,000 neurons, 20 million synapses, and 25 neuronal types. The optimized network reproduces population firing statistics and oscillatory dynamics and generates spatially selective hippocampal activity without explicit place-field tuning. The model achieves a runtime factor of approximately 20 relative to biological time, with scaling analysis predicting approximately 50 at full scale, supporting simulations over behavioral timescales. This framework provides a scalable approach for constructing and functionally optimizing anatomically constrained neural circuit models.

